# 16^th^ IHIW: Global analysis of registry HLA haplotypes from 20 Million individuals: Report from the IHIW Registry Diversity Group

**DOI:** 10.1111/iji.12031

**Published:** 2012-12-26

**Authors:** M Maiers, L Gragert, A Madbouly, D Steiner, S G E Marsh, P-A Gourraud, M Oudshoorn, H Zanden, A H Schmidt, J Pingel, J Hofmann, C Müller, H-P Eberhard

**Affiliations:** * Bioinformatics Research, National Marrow Donor Program (NMDP)Minneapolis, MN, USA; † Czech Stem Cells Registry (CSCR)Prague, Czech Republic; ‡ Anthony Nolan Research Institute (ANRI) and UCL Cancer InstituteLondon, UK; § Registre France Greffe de MoelleParis, France; ¶ Bone Marrow Donors Worldwide (BMDW), Europdonor FoundationLeiden, the Netherlands; ** Deutsche Knochenmark-spenderdatei (DKMS)Tuebingen, Germany; †† Zentrales Knochenmarkspender-Register (ZKRD)Ulm, Germany

## Abstract

This project has the goal to validate bioinformatics methods and tools for HLA haplotype frequency analysis specifically addressing unique issues of haematopoietic stem cell registry data sets. In addition to generating new methods and tools for the analysis of registry data sets, the intent is to produce a comprehensive analysis of HLA data from 20 million donors from the Bone Marrow Donors Worldwide (BMDW) database. This report summarizes the activity on this project as of the 16IHIW meeting in Liverpool.

## Introduction

The ultimate goal of the Registry Diversity Project has been to deliver a global analysis of HLA haplotype frequencies from the data of the worldwide registries of Bone Marrow Donors Worldwide (BMDW). These data are important for recruitment and matching processes leading to the procurement of unrelated haematopoietic stem cell (HSC) donors.

Haplotype frequency estimation (HFE) algorithms have been used for decades to describe the HLA diversity of a population (Ceppellini *et al*., 1955; Yasuda & Kimura, 1968; Piazza, 1975; Yasuda, 1978; Morton *et al*., 1983; Hawley & Kidd, 1995; Long *et al*., 1995; Salem *et al*., 2005). Haplotype analysis from HLA data has been carried out in the Anthropology components of International Histocompatibilty Workshops since the early days of HLA. Registry data sets provide unique challenges to these algorithms due to the size, number of loci and typing resolution heterogeneity of each data set. Despite the existence of a number of implementation of the EM algorithm, none of the publically available implementations are able to deal with the sample size, ambiguity, missing data and nuances of the HLA nomenclature (Müller *et al*., 2003; Kollman *et al*., 2007; Lancaster *et al*., 2007; Maiers *et al*., 2007; Eberhard *et al*., 2010; Excoffier & Lischer, 2010; Schmidt *et al*., 2011).

Through extensive experimentation with HSC registry data and comparison of HFE methods after the 14IHIW and 15IHIW, the registry diversity working group identified the need for validating specific aspects of this task within a controlled environment for the accurate assignment of the parameters affecting HFE methods, as well as to quantify the error.

Three principal tasks were outlined for the 16IHIW project:

1. *Estimation of the accuracy of HFE on simulated data sets with deviation from Hardy–Weinberg Equilibrium*: Registry data sets are not assembled by sampling from genetically distinct randomly mating populations. Often these data sets have deviation from Hardy–Weinberg Equilibrium (HWE). To address this issue, we developed a series of simulations that produce populations deviating from HWE in specific ways. By carrying out controlled experiments on these simulated data sets, we will be able to develop robust methods for addressing this issue and deal with it appropriately with real data.
2. *Estimation of the accuracy of methods of estimating high-resolution haplotypes from large data sets of mixed resolution*: All HLA typing methods include some level of ambiguity. DNA methods are typically reported in terms of allelic ambiguity, but there are other forms of ambiguity: genotypic (inability to set phase), historical (typing to a fixed snapshot of a growing allele list) and ambiguity due to incomplete definition of reference alleles (missing sequence). The ultimate form of ambiguity is the lack of typing data. The clinical use of HSC products involves matching at high-resolution (HR) across five HLA loci (HLA-A, -B, -C, -DRB1 & -DQB1). Yet the majority of the HLA typing data is not at this level. Although several groups have implemented algorithms to perform HFE in the context of ambiguous and incomplete data, we have the need to cross-validate such methods and also to quantify the error for different levels of difficulty.
3. *Worldwide donor registry analysis: high-reslution HLA A-C-B-DRB1-DQB1 haplotype frequencies for Bone Marrow Donors Worldwide (BMDW) registries*: When this project was initiated, all registries submitting data to BMDW were invited to opt in or out of participation in a comprehensive HLA haplotype frequency analysis. This task represents the culmination of this project: haplotype frequencies will be generated for all registries of the BMDW. Armed with the results of the previous tasks in this workshop (and the work from the previous two workshops), the results will be generated with confidence in the quality of the output and clear indication of the sources and magnitude of error. We expect these data will be of tremendous value both within the HSC transplant community and beyond.

This report outlines the experiments developed through the collaborative efforts of this working group. Although the IHIW meeting was productive in terms of definition of the approach, the majority of the work still lies ahead.

## Estimation of the accuracy of HFE on simulated data sets with deviation from HWE

To obtain an adequate estimate of the accuracy of HFE under deviation from HWE, we developed three series of data sets based on simulations that produce populations that deviate from HWE in specific ways.

A general simulation framework was developed for the 15th IHIW, which involves the creating of a global population (GP) of haplotypes and a series of sample populations (SP) of diploid individuals, each of which was generated by independently drawing two haplotypes from the pool of A-B-DRB1 low-resolution haplotypes. This process is repeated to produce a set of K sample populations of a particular size N, each having a directly countable distribution of haplotype frequencies *HF*(SP_1_) *… HF*(SP_K_). Haplotype frequency estimation is performed on these same sample populations to generate *HFE*(SP_1_) *… HFE*(SP_K_). From here, it is possible to explicitly measure the error due to sampling, the error due to estimation and the combined error.

### Excess heterozygosity/homozygosity simulation

In the first set of experiments, we have used the simulation framework to create sample populations that are in HWE. Due to the nature of the random sampling process, the sample populations did not deviate from HWE. Now haplotype pairs are reshuffled to create a deviation from HWE by adding different proportions of homozygotes and heterozygotes under a frequency-invariant transformation. This process literally identifies two pairs of individuals whose haplotypes are swapped in a symmetric way such that there is an increase (or decrease) in homozygosity with no deviation in haplotype frequencies. Departure from HWE is correlated with the number of times these processes are replicated. Although this is an artificial method for modifying the population, it is an important first experiment because all other variables are held fixed (N, GP, HF_i_), and the impact of deviation from HWE on HFE can be directly measured by comparing HF_i_ and HFE_i_ to each other. For these experiments, we chose *K* = 10 and *N* = 100 000.

### Wahlund effect simulation framework

This framework simulates deviation from HWE in a more biologically realistic way: the reduction of heterozygosity in a population caused by subpopulation structure with different allele frequencies.

**Figure 1:**
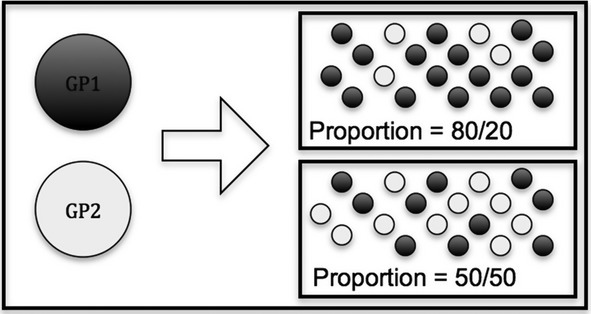
Wahlund effect simulation: each sample population is constructed with individuals where the proportions specify the population origin from two different global populations. Individuals have either two haplotypes from GP1 or two haplotypes from GP2. GP, global population.

The simulation framework is based on two global populations (GP_1_ and GP_2_). For these experiments, we are generating *K* = 10 replicates each of size *N* = 100 000 across a series of nine population proportions: 10/90, 20/80, …, 90/10. For each of these proportions, the sample population (SP) contains individuals derived from either one or the other of the two global populations (Fig. 1). The challenge for haplotype estimation here is to produce haplotype frequency estimates for both populations (HFE1 and HFE2).

Two full sets of simulations were run: one where GP1 and GP2 have no significant overlap (data where the underlying GPs are derived from registries in Poland and Taiwan) and a more difficult case where there is significant overlap of haplotypes (data where the GPs are derived from registries in Germany and France).

### First-generation admixture simulation framework

**Figure 2:**
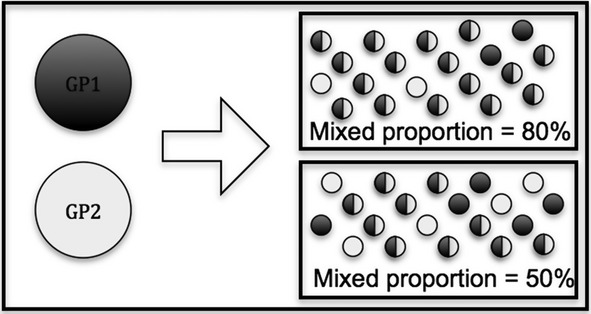
First-generation admixture simulation: each sample population is constructed with a given proportion of individuals with one haplotype from GP1 and the other GP2 and the remaining proportion with equal number having both haplotypes in GP1 or GP2. GP, global population.

This framework simulates a more complicated situation where, for a portion of the individuals, its two haplotypes are drawn from two different populations (GP1 or GP2) at random. This proportion varies from 10%, 20%, …, 90% with the remainder of the sample composed of equal portions of individuals with both haplotypes from GP1 or both haplotypes from GP2 (Fig. 2). For example, in the case where the admixed proportion is 80% with one haplotype from each of GP1 and GP2, there are 10% with both haplotypes drawn from GP1 and 10% with both haplotypes drawn from GP2. With this scheme, the 50% admixed case is the only one where the genotypes would be expected to conform to expectations under HWE relative to the mean of the haplotype frequencies of GP1 and GP2.

These experiments use *K* = 10 replicates of size *N* = 100 000. Again here, the challenge for haplotype estimation is to produce haplotype frequency estimates for both populations (HFE1 and HFE2) and also from source populations where there is or is not significant overlap (Poland & Taiwan and Germany & France).

The data sets for all three experiments have been developed and are currently under analysis.

## Estimation of the accuracy of methods of estimating high-resolution haplotypes from large data sets of mixed resolution

We designed experiments to address two issues relating to HFE in large registry data sets, which are HLA typing resolution (including missing data) and the impact of the underlying population diversity.

### Addressing HLA typing ambiguity: untyped loci and variation in typing resolution

**Figure 3:**
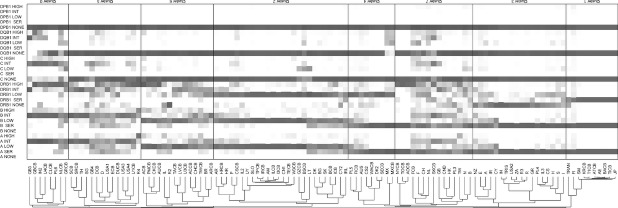
Two-dimensional clustering of Bone Marrow Donors Worldwide (BMDW) registries based on characteristics of HLA typing resolution and loci typed. Darker colours indicate larger proportions. Columns sum to unity.

We defined a set of variables to describe the HLA typing ‘profile’ for all registries in BMDW. This involved determining the proportion of donors typed at five levels of resolution: high, intermediate, low, serology and not-typed across six HLA loci: HLA-A, -B, -C, -DRB1, -DQB1 and -DPB1. We then clustered the registries according to these profiles and determined six general categories to study (Fig. 3). For each category, a representative registry was identified (BMDW codes: D, IL, NL, PL6, SG, TW) as a prototype for each category.

The simulation experiment here involves using a GP of HR full-haplotype frequencies to generate a series of sample populations (*K* = 100 replicates of *N* = 100 000 individuals) for six different HLA typing/resolution profiles intended to match the proportion of the six representative registries in each group.

To simulate different levels of typing resolution, we developed a system to introduce typing ambiguities by producing the corresponding genotyping at lower resolution for each sample. For simplicity, we implemented four of the five levels of resolution per locus:

- DNA at the allele-family (Low), e.g. A*02
- DNA SSO using a contemporary typing kit (Intermediate), e.g. A*02:AB
- DNA SBT considering only genotypic ambiguity in the exons encoding the peptide-binding domains (High), e.g. A*02:02
- No typing (e.g. HLA-C).

The experiment is designed to evaluate the impact of typing resolution on haplotype frequency estimation by comparing the output (HFE) to the sample frequencies (HF) and also the GP.

The framework for generating simulated typing results is still in development.

### Measurement of population diversity

Another factor that affects the quality of HFE in registry data sets is the underlying frequency distribution. The more diverse the underlying population the larger the effect of sampling error on the frequencies becomes. Several methods were proposed to measure the diversity of haplotype frequency distribution for different populations.

**Figure 4:**
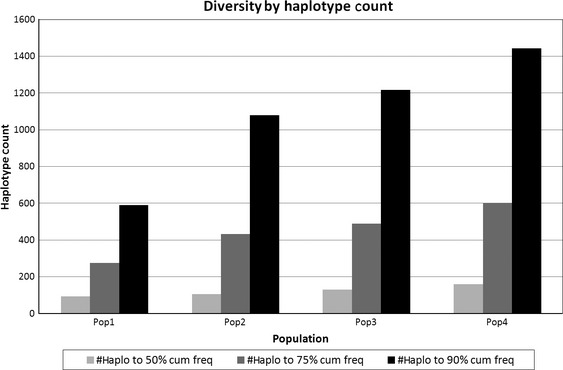
Population diversity by haplotype count to reach 50%, 75% and 90% cumulative frequency.

Figure 4 displays the diversity of four BMDW populations measured by the haplotype count to reach cumulative frequencies of 50%, 75% and 90% of the total haplotype frequency. Pop1 shows the least diversity where only 589 haplotypes are needed to reach 90% cumulative frequency while it takes 1442 haplotypes to reach the same value for pop4, which is the most diverse in this plot. The frequencies were all generated using an equal sample size of 75 000 samples.

The goals of these analyses is to understand the relationship between the shapes of the frequency distribution, parameterized by qualitative statistics or by fitting it to an exponential or power law distributions, and then determining, by regression, the interaction between the frequency distribution and sampling error.

## Worldwide donor registry analysis: high-resolution HLA-A-C-B-DRB1-DQB1 haplotype frequencies for BMDW registries

**Figure 5:**
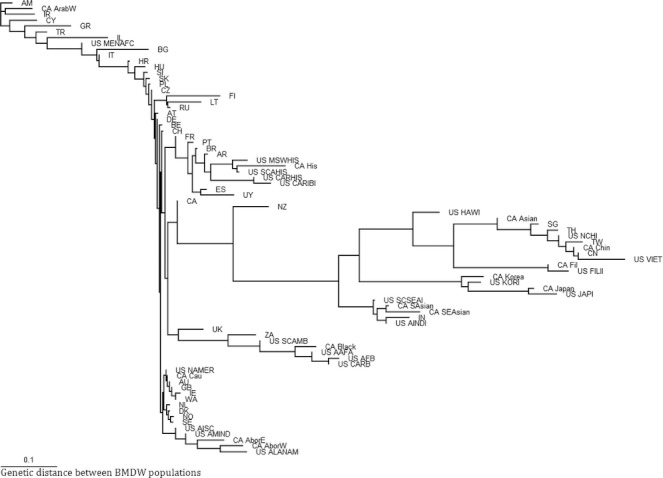
Nei's Genetic Distance on haplotype frequencies for Bone Marrow Donors Worldwide (BMDW) populations.

A challenge with estimating HR HLA-A-C-B-DRB1-DQB1 haplotypes in BMDW is that HR HLA typing is not available from some registries. Also HLA-C and HLA-DQB1 typing data are partially or completely missing. To handle these issues and generate operationally useful haplotype frequencies, a mapping scheme was developed as follows:

Preliminary haplotype frequencies were generated for all registries in BMDW that agreed to participate in this project at low-resolution for HLA-A-B-DRB1. Nei's genetic distance (Nei, 1972) was used to analyse these frequencies to determine similarity between populations (Fig. 5) for use in the following mapping procedures:

1. Determine the ‘222’ A-B-DRB1 haplotype frequencies for the registry to be mapped (222 refers to the first field of the HLA allele, e.g. A*02-B*07-DRB1*13).
2. For each 222 haplotype, get a list of matching 444 haplotypes from a neighbouring population (based on genetic distance) that has suitable HR data (444 refers to the first two fields of the HLA allele, e.g. A*02:02-B*07:02-DRB1*13:01).
3. Split the 222 frequencies proportionally using the relative frequency from the matching 444 haplotypes from the other frequency data set.

The results of this genetic distance analysis and the resulting population dendogram (using the PHYLIP nearest-neighbor algorithm) is presented in Figure 5. As a preliminary result this tree provides clear evidence of population separation and clustering of continental and sub-contiential groups (Eastern Europe, Africa, East Asia, etc.). A similar procedure was used to deal with missing HLA-C and HLA-DQB1 data. We created a framework to validate some of the frequencies generated by the above method. The validation process involved separating the HR subsets of the samples used to generate the frequencies. Allelic ambiguities were then artificially introduced to the HR samples bringing the typing resolution down to the two-digit (family typing) level. Ambiguous typings were then input to an HLA imputation process to infer the possible HR assignments that correspond to the ambiguous input, and the results were compared to the original HR data. The choice of registries participating in the validation study was bound by the provided HR sample size.

Unfortunately, most registries did not have an adequate HR sample size for the C and DQB1 loci, which constrained the validation to three-locus (A-B-DRB1) frequencies where the largest sample size was available. Frequencies were validated for five BMDW registries. The validation framework included comparing expected and actual predictions using weighted city block distance, receiver-operating characteristics, per cent of maximum a posteriori prediction probability and average probability of correct predictions. Disparities were found between the different frequencies based on the quality of the data used to generate the frequencies. In the future, we hope to expand the validation framework and include more registries as better quality and bigger samples become available. Including data from confirmatory typing performed on registry donors is critical to the validation process for these data.

## Discussion

The goal of the IHIW registry diversity working group is the comparison, validation and improvement of bioinformatics methods and tools for HLA haplotype frequency analysis specifically designed to address issues of blood stem cell registry data sets. The distinguishing features of these data sets are:

- Very large samples
- Heterogeneous typing resolution and methods
- Missing data for one or more loci
- Ethnic diversity (self-identified and unidentified)
- Population sub-structure determination (clustering)
- Hardy–Weinberg deviation (nonrandom mating).

The global collaboration developed via this working group has been essential for addressing this challenge both in terms of bringing together the data and also in collaborating to design a series of experiments that will be critical in order to ensure the quality of the frequency data we generate.

The goal for these data is to apply them to projecting registry growth and the effects on matching. Other immediate uses of these data are:

- Genetic distance analysis to determine the relatedness of donor registries
- A ‘dictionary of haplotypes’ and tools for delivery (such as haplostats.org)
- Use for donor search
- Informing registry recruitment strategies
- A publically available resource for science

## Conclusion

This project has been a joint activity of the IHIW and the World Marrow Donor Association (WMDA). The WMDA and its member registries have a vested interest in this analysis as it is integral to the effort of registry development and matching. This component began with the 14th IHIW and continued through the 16th IHIW culminating with a series of experiments that were carried out to look at the impact of missing data, typing ambiguity and bias in simple, but practical, situations (manuscript in preparation). This meeting has generated a roadmap for how to move forward on these challenges.
